# Host genetics and pathogen species modulate infection-induced changes in social aggregation behaviour

**DOI:** 10.1098/rsbl.2022.0233

**Published:** 2022-08-31

**Authors:** Valéria Romano, Amy Lussiana, Katy M. Monteith, Andrew J. J. MacIntosh, Pedro F. Vale

**Affiliations:** ^1^ IMBE, Aix Marseille Univ., Avignon Univ., CNRS, IRD, Marseille, France; ^2^ Institute of Evolutionary Biology, School of Biological Sciences, University of Edinburgh, Edinburgh, UK; ^3^ Kyoto University Wildlife Research Center, Japan

**Keywords:** social aggregation, infection avoidance, sickness behaviour, genetic variation, pathogen dose, bacterial infection

## Abstract

Identifying how infection modifies host behaviours that determine social contact networks is important for understanding heterogeneity in infectious disease dynamics. Here, we investigate whether group social behaviour is modified during bacterial infection in fruit flies (*Drosophila melanogaster*) according to pathogen species, infectious dose, host genetic background and sex. In one experiment, we find that systemic infection with four different bacterial species results in a reduction in the mean pairwise distance within infected female flies, and that the extent of this change depends on pathogen species. However, susceptible flies did not show any evidence of avoidance in the presence of infected flies. In a separate experiment, we observed genetic- and sex-based variation in social aggregation within infected, same-sex groups, with infected female flies aggregating more closely than infected males. In general, our results confirm that bacterial infection induces changes in fruit fly behaviour across a range of pathogen species, but also highlight that these effects vary between fly genetic backgrounds and can be sex-specific. We discuss possible explanations for sex differences in social aggregation and their consequences for individual variation in pathogen transmission.

## 1. Background

Understanding how infection modifies group behaviour, thereby altering social connectivity and transmission dynamics, is a central focus of infectious disease research [1–5]. We can consider several types of behavioural responses to infection [6,7]. Infection avoidance is the first line of behavioural defence, where hosts modify their behaviour if they perceive an infection risk in their environment or from conspecifics [8–11]. This may include spatial or habitat avoidance [12,13], trophic avoidance [11,14,15] and social avoidance [11,16]. Nevertheless, it is rarely possible to completely avoid infection, as many common infection routes involve activities that are central to organismal physiology and fitness, including foraging and feeding. Once infected, as part of a generalized sickness response, individuals may actively self-isolate or due to their lethargic behaviour, engage in fewer social interactions [17–19], while uninfected individuals may also actively avoid those showing signals of infection [8,9,20]. Altogether, this variation in social behaviour drives the likelihood of pathogen transmission [2,21].

The extent to which hosts modify their behaviour during infection is likely to depend on their environmental and social contexts [22–24], as well as on host and pathogen genetic factors [25–27]. For example, following an immune challenge, isolated zebra finches show reduced activity, but those kept in a colony setting do not [18], while in fruit flies, social aggregation and infection risk varies according to the sex ratio of the group [23]. It is therefore important to investigate the effect of different sources of variation in infection-induced changes in insect social behaviour. The fruit fly *Drosophila melanogaster* is particularly powerful model to address this question due to its genetic tractability and its extensive use as a model of host–pathogen interactions and behavioural ecology and genetics [22,27–29]. For example, social behaviour in *D. melanogaster* shows moderate heritability and responds to directional selection [22,27]. Here, we investigate how the behavioural response to infection in *Drosophila* is modified by pathogen species and infectious dose, or host genetic background and sex.

In one experiment, we focus on pathogen sources of variation and ask how social aggregation behaviour changes over time when flies are exposed to either low or high doses of different bacterial pathogens. We used social groups comprised of both infected and susceptible individuals, which allowed us to test how infection affects the behaviour of infected flies, how the presence of infected flies affects the behaviour of susceptible flies, and whether there is any evidence that healthy flies show avoidance behaviour towards infected conspecifics. In a separate experiment, we inquire how host genetic background generates differences in social aggregation following infection, and how these effects differ between males and females.

## 2. Material and methods

### (a) Fly lines

In experiment 1 (pathogen variation), we used female flies from a large outbred population, originally derived from DGRP (*Drosophila* Genetic Reference Panel). In experiment 2 (host variation), we used male and female flies from 10 DGRP lines (RAL-208, RAL-852, RAL-427, RAL-304, RAL-21, RAL-375, RAL-28, RAL-324, RAL-358, RAL-712) selected to include a range of sociality scores [30]. Detailed rearing conditions are provided in the electronic supplementary material.

### (b) Bacterial strains and culture

In experiment 1, we established systemic infections with one of four species of bacterial pathogen with well-described pathology in *D. melanogaster*: *Enterococcus faecalis*, *Pseudomonas entomophila*, *Serratia marcescens* DB11 and *Providencia rettgeri*. In experiment 2, we used a single bacterial fly pathogen, *P. entomophila*. Detailed culture conditions are provided in the electronic supplementary material.

### (c) Experiment 1 (pathogen variation)

Social interaction chambers consisted of 50 mm Petri dishes containing 8% sugar-agar medium. In total, we set up 24-replicate social groups for each pathogen and dose (*N* = 192), plus 24 control groups. Flies were anaesthetized using light CO_2_ and infected in the mesopleuron with one of four bacterial pathogens at OD 0.1 or 0.01 using a 0.14 mm diameter stainless steel pin. Control flies received an equivalent inoculation with sterile LB. The experiment was blocked over 4 consecutive days (10.00–14.00), with chambers including all treatments spread across each block. Each Petri dish contained six uninfected, susceptible female flies and six female flies infected with a specific bacterial pathogen at a specific dose. Infected flies were marked with red fluorescent powder on the prothorax and the underside of the abdomen using a cotton bud (electronic supplementary material, figure S1). Control plates were also set up containing 12 uninfected individuals, with half marked as above. Flies were allowed an hour of recovery from the systemic infection and marking before being re-anaesthetized using light CO_2_ and added to the social interaction chambers. Thirty minutes were allowed for habituation before photos of the groups were taken every 30 min until 4 h post-infection. Pictures were processed in ImageJ, to estimate coordinates of each individual. Social aggregation was then measured using the *pairdist* function in the *spatstat* package in R [31] (electronic supplementary material, figure S2). The pairwise distance between each pair of flies within a dish was used to calculate three sociality metrics per dish: (i) the mean pairwise distance between infected flies, which is relevant to evaluate changes in aggregation due to sickness behaviour; (ii) the mean pairwise distance between susceptible flies, and (iii) the mean pairwise distance between infected and susceptible flies, which enables testing whether susceptible flies tend to avoid infected flies, when compared to the control group. Therefore, each dish resulted in two intra-class measures (within infected and within susceptible) and one inter-class measures (between infected and susceptible).

### (d) Experiment 2 (host variation)

For each of the 10 fly lines, we set up single-sex groups of flies, divided into infected and control, and each fly line–sex–treatment was replicated 11–12 times, for a total of 466 social aggregation assays. Each group consisted of 12 flies systemically infected with *P. entomophila* (or sterile LB medium for uninfected control groups) using a stainless pin. Following infection, flies were lightly anaesthetized with CO_2_ and transferred to 55 mm Petri dishes containing agar. After a 30-min habituation period, one set of photographs were taken. Here we used the median nearest neighbour distance (NND) of each group as a measure of social aggregation [25,30]. Individual fly positions in each image were marked in the middle of the fly thorax using Fiji (**F**iji **I**s **J**ust **I**mageJ), and the nearest neighbour distances between each pair of flies was calculated using the ‘NND’ plugin within the software Fiji [32].

### (e) Statistical analysis

All raw data and analysis R code are available at https://doi.org/10.5281/zenodo.6554320 [33]. Data from experiment 1 were analysed using linear mixed effects models, separately for each social class (i.e. within infected, within susceptible, between infected and susceptible). We used the mean pairwise distance as the response factor, pathogen, dose and time as predictor variables, and day of assay as a random effect. For experiment 2, we used a linear mixed effects model with the log_10_ of median NND as the response variable, line, sex and infection status as predictors, and day of the assay as a random effect. All possible interactions between line, sex and infection status were included. A more detailed description of the analysis can be found in the electronic supplementary material.

## 3. Results

### (a) Pathogen drivers of social aggregation

#### (i) Intra-class infected

**Figure 1.**
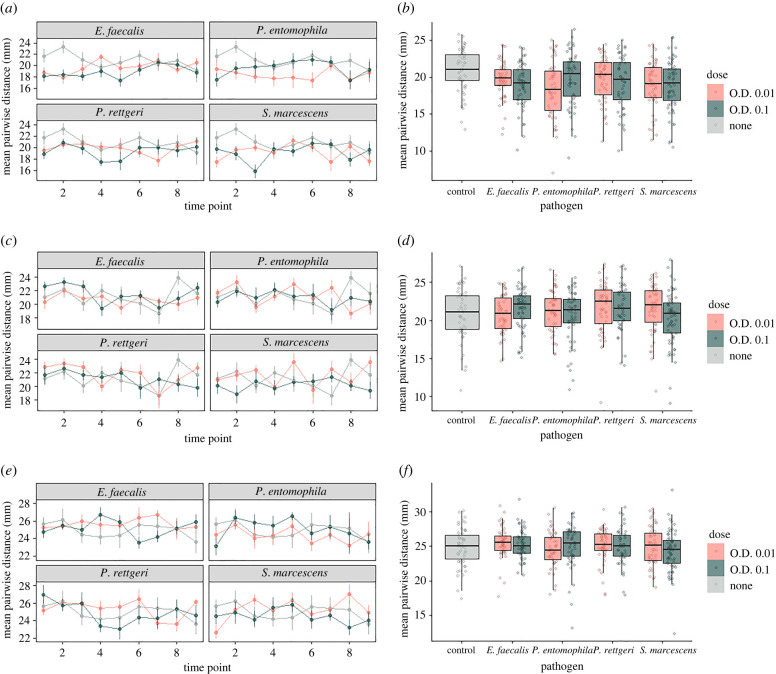
Mean pairwise distance in millimetres (mm) when considering (*a*,*b*) intra-class distance within infected flies, (*c*,*d*) intra-class distance within susceptible flies, (*e*,*f*) inter-class distance between infected and susceptible flies, of both low (O.D. 0.01) and high (O.D. 0.1) doses. (*a*,*c*,*e*) The mean pairwise distance (mm) ± s.e. (*b*,*d*,*f*) The mean pairwise distance (mm) for each pathogen and dose, averaged across all time points. Time points refer to the interval of data collection: nine pictures taken every 30 min post-infection. Intra-class infected flies aggregated significantly closer than control flies (*b*).

Our analysis showed a significant effect of pathogen species on the mean pairwise distance within infected flies, with a non-significant trend for an interaction between dose and pathogen (table 1 and figure 1*a,b*). This trend is likely driven by flies infected with low dose (OD = 0.01) of *P. entomophila* (electronic supplementary material, table S1; *p* = 0.0005) and high dose (OD = 0.1) of *E. faecalis* (*p* = 0.005) and *S. marcescens* (*p* = 0.04) aggregating closer together when compared with control uninfected flies. When comparing the overall rate of social aggregation within infected flies to uninfected control flies, we observed that infection with almost all tested pathogens resulted in a reduction in mean pairwise distance when compared to controls: low dose (OD = 0.01) = 1.25 mm for *E. faecalis* (post-hoc Dunnett's test, *p* ≤ 0.05), 2.61 mm for *P. entomophila* (*p* < 0.001), 1.05 mm for *P. rettgeri* (*p* = 0.11) and 1.78 mm for *S. marcescens* (*p* < 0.01). High dose (OD = 0.1) = 2.19 mm for *E. faecalis* (< 0.001), 1.47 mm for *P. entomophila* (*p* ≤ 0.05), 1.63 mm for *P. rettgeri* (*p* ≤ 0.01) and 1.76 mm for *S. marcescens* (*p* < 0.01).

**Table 1.** Outputs for ANOVA performed on social aggregation testing (A) intra-class pairwise distance within infected flies, (B) intra-class pairwise distance within susceptible flies, (C) inter-class pairwise distance between infected and susceptible flies.

	*F*	d.f.	*p*-value
(A) intra-class infected			
pathogen	4.501	4	0.001
dose	0.782	1	0.377
time	0.276	1	0.6
pathogen × dose	2.568	3	0.053
pathogen × time	1.373	4	0.241
dose × time	0.597	1	0.44
pathogen × dose × time	0.123	3	0.947
(B) intra-class susceptible			
pathogen	0.959	4	0.429
dose	2.084	1	0.149
time	6.192	1	0.013
pathogen × dose	3.38	3	0.018
pathogen × time	1.757	4	0.135
dose × time	0.184	1	0.668
pathogen × dose × time	0.303	3	0.823
(C) inter-class infected-susceptible			
pathogen	1.345	4	0.251
dose	1.972	1	0.161
time	1.728	1	0.189
pathogen × dose	1.87	3	0.133
pathogen × time	1.463	4	0.211
dose × time	2.488	1	0.115
pathogen × dose × time	1.064	3	0.364

#### (ii) Intra-class susceptible

Among the subgroup of susceptible flies, we observed a reduction in the pairwise distance over the course of the experiment (table 1, time effect, *p* = 0.013) and an interaction between dose and pathogen (table 1, *p* = 0.018, figure 1*c,d*). We did not observe any difference between the overall aggregation pattern of susceptible flies when compared to control groups: *E. faecalis* (electronic supplementary material, table S1; post-hoc Dunnett's test, OD = 0.01: *p* = 0.98; OD = 0.1: *p* = 0.65), *P. entomophila* (OD = 0.01: *p* = 0.96; OD = 0.1: *p* = 1), *P. rettgeri* (OD = 0.01: *p* = 0.32, OD = 0.1: *p* = 0.97) and *S. marcescens* (OD = 0.01: *p* = 0.59, OD = 0.1: *p* = 0.42).

#### (iii) Inter-class infected-susceptible

We did not find any effect of pathogen, dose and/or time when testing the inter-class distance between infected and susceptible flies (table 1 and figure 1*e,f*), providing no evidence of social avoidance between susceptible and infectious flies in our experiments.

### (b) Host drivers of social aggregation

**Figure 2.**
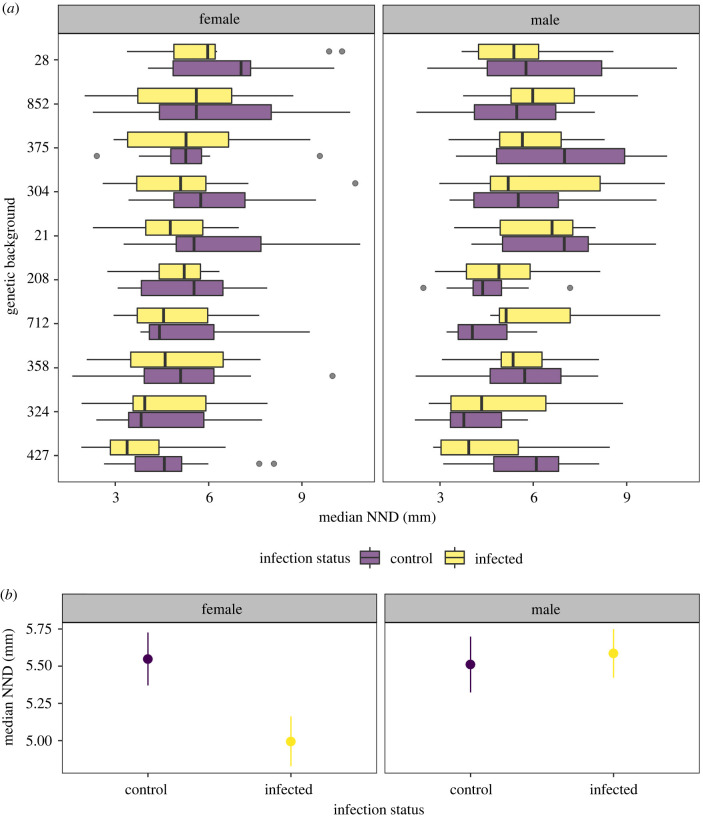
(*a*) Box plots showing the NND in millimetres (mm) for males and females (uninfected and infected) among DGRP lines. Grey data points indicate outliers. (*b*) Plot of infection status and sex, based on median NND in millimetres (mm). Females, but not males, aggregate more closely following infection.

In a second experiment, we tested whether social aggregation following systemic *P. entomophila* infection differs between flies of different genetic backgrounds and sex. We found that social aggregation is explained by host DGRP line (figure 2*a* and table 2, Line effect, *p* = 0.001) and that patterns of social aggregation differed between males and females (table 2, sex effect, *p* = 0.03). We also observed a significant interaction between sex and infection status (table 2, *p* = 0.026, figure 2*b*). While male and female flies have near identical NND aggregation in the absence of infection (*p* = 1, least-square means, *t* = 0.15), infected females aggregated more closely than infected males by 1.15 mm (electronic supplementary material, table S2; *p* = 0.01, *t* = −3.04, figure 2*b*). This sex difference in post-infection aggregation was observed regardless of DGRP line (there was no significant line × sex × infection interaction, table 2).

**Table 2.** Output for ANOVA performed on social aggregation testing the influence of male and female flies of 10 DGRP lines.

	*F*	d.f.	*p*-value
line	4.676	9	<0.0001
sex	4.758	1	0.03
infection status	1.756	1	0.186
line × sex	1.05	9	0.399
line × infection status	1.448	9	0.166
sex × infection status	4.959	1	0.026
line × sex × infection status	0.626	9	0.775

## 4. Discussion

Social avoidance of infection is a widespread mechanism of defence in the animal kingdom [9,34]. Sick individuals may decrease social connectivity due to lethargic behaviour or actively self-isolate [16,35], but they can also be avoided by healthy individuals to avoid direct routes of infection [9,20,36]. This social behavioural flexibility leads to detectable changes in the group social structure, which affects the risk of contagion among individuals [2,37]. In this study, we observed increased aggregation (shorter distances) within female infected flies—which may be due to a sickness response—but we did not find evidence that infected and susceptible flies tend to avoid each other. Given males have a body length of 1.5–2.5 mm, while females are slightly larger (1.7–3 mm) [25], the largest effects we found mean flies would be nearly a full body length closer to each group member.

Distinct ways of modifying social aggregation have been described in different social insects and may occur due to host's social context (e.g. sex ratio, [23], alteration of feeding patterns [15], or changes in oviposition site choice [11]). An additional source of changes in infected host behaviour, which we did not explore in the current study, is that pathogens can often manipulate the behaviour of their hosts to increase the likelihood of transmission [7,38,39]. One relevant example relates to the increased production of attraction pheromones in flies infected with *P. entomophila*, resulting in increased aggregation between healthy and infected flies [40]. It is unclear if the increased aggregation in females we observed could have been mediated by similar pathogen-derived effects.

Regarding sex-specific aggregation during infection, these appear to be pathogen specific. While this study found increased aggregation of female flies infected with pathogenic bacteria (relative to no change in males), other work identified sex differences in the opposite direction during virus infection, where males infected with *Drosophila* C virus aggregated further apart, with no apparent change in female social behaviour following DCV infection [25]. A recent analysis of 59 F_1_-hybrids derived from the DGRP panel (the same panel of flies used here) also reported little correlation between the sociability of male and female flies [27].

One possible explanation is that sex differences are a consequence of sex-based costs of social aggregation [41–43]. Given that males usually display costly aggressive behaviours [44], avoiding aggregating closely when infected may also avoid the costs of aggressive encounters, while saving resources for immune deployment [45]. Female flies, however, employ generally less costly aggressive behaviours [46,47]. Differences in social aggregation costs could therefore explain why infected females aggregate more closely than males, and maintaining or augmenting sociality during infection has been suggested to reduce the impact of infection in some systems [4].

We also found that genetic background strongly influences social aggregation in fruit flies. This result confirms previous findings [25,27,30], where sociality *in D. melanogaster* exhibits moderate broad sense heritability (*H*^2^ = 0.21–0.24) [27], and responds readily to directional selection [48]. This large variation is to be expected for a polygenic trait such as sociality [27], and is not just characteristic of insects, as genetic background has been also found to influence social behaviours in humans and other mammalian species [9,49].

In summary, we find that flies modify their social behaviour following bacterial infection. These differences were pathogen and dose dependent, and for at least one pathogen species, this response was sexually dimorphic, with infected females aggregating more closely than infected males. Our work therefore contributes to further our understanding of this important driver of infection dynamics and of the ecology and evolution of both hosts and pathogens [2,4,36].

## Data Availability

All raw data and analysis R code are available at https://doi.org/10.5281/zenodo.6554320 [33]. Electronic supplementary material is available online [50].
